# Social media & psychiatry: Research opportunities.

**DOI:** 10.1192/j.eurpsy.2023.186

**Published:** 2023-07-19

**Authors:** M. A. Alvarez-Mon

**Affiliations:** ^1^Hospital Universitario Infanta Leonor, Madrid; ^2^University of Alcalá, Alcala de Henares, Spain

## Abstract

**Abstract:**

In the field of health, social networks are increasingly used for research since they have demonstrated multiple uses. Proof of this is that more and more projects financed by public entities use this methodology, as well as scientific publications.

In the symposium, how to do this type of research will be explained in a practical and useful way for the listener. It will be explained with practical examples, and based on successful publications, how this type of work is designed, what type of people should integrate this research team, how to carry out the project, interpret the results and publish them.

We will also address the difficulties encountered and how to overcome them. Attendees will be able to ask all the questions they deem appropriate.

**Disclosure of Interest:**

None Declared

